# Benign perimesencephalic hemorrhage occurring after previous aneurysmal subarachnoid hemorrhage: a case report

**DOI:** 10.1186/1752-1947-4-405

**Published:** 2010-12-14

**Authors:** Richard H Singleton, Dean B Kostov, Hilal A Kanaan, Michael B Horowitz

**Affiliations:** 1 Department of Neurological Surgery, University of Pittsburgh Medical Center, Suite B-400, 200 Lothrop Street, Pittsburgh, PA 15213, USA

## Abstract

**Introduction:**

Both aneurysmal subarachnoid hemorrhage and benign perimesencephalic hemorrhage are well-described causes of spontaneous subarachnoid hemorrhage that arise as a result of different pathologic processes. To the best of the authors' knowledge, there have been no reports of both vascular pathologies occurring in the same individual.

**Case presentation:**

A 51-year-old Caucasian woman with a history of aneurysmal subarachnoid hemorrhage presented five years after her initial treatment with ictal headache, meningismus, nausea and emesis similar to her previous bleeding event. Computed tomographic imaging revealed perimesencephalic bleeding remote from her previously coiled anterior communicating artery aneurysm. Both immediate and delayed diagnostic angiography revealed no residual filling of the previously coiled aneurysm and no other vascular anomalies, consistent with benign perimesencephalic hemorrhage. The patient had an uneventful hospital course and was discharged to home in good condition.

**Conclusions:**

This report for the first time identifies benign perimesencephalic hemorrhage occurring in the setting of previous aneurysmal subarachnoid hemorrhage. The presence of a previously treated aneurysm can complicate the process of diagnosing benign perimesencephalic hemorrhage. Fortunately, in this case, the previously treated anterior communicating artery aneurysm was remote from the perimesencephalic hemorrhage and could be ruled out as a source. The patient's prior aneurysmal subarachnoid hemorrhage did not worsen the anticipated good outcome associated with benign perimesencephalic hemorrhage.

## Introduction

Spontaneous subarachnoid hemorrhage (SAH) is a significant clinical problem that occurs most commonly as a result of aneurysm rupture. In approximately 15% of cases, however, no aneurysm can be identified by cerebral angiography. Although in a minority of cases occult aneurysms are eventually identified, non-aneurysmal SAH represents an interesting clinical problem that can occur as a result of many different pathologies, including vasculitis, arterial dissection, intra-cranial or cervical arteriovenous malformation or fistula, clotting diatheses, antiplatelet and/or anticoagulant medication, pituitary apoplexy and tumors [1]. Benign perimesencephalic hemorrhage (BPH) is another described type of non-aneurysmal SAH and is thought to account for approximately one- to two-thirds of non-aneurysmal SAH and 5-10% of SAH as a whole [1,2]. The presenting symptoms of both aneurysmal SAH and BPH overlap and include sudden onset "thunderclap" headache, nausea, emesis and meningismus. The diagnosis of BPH can be made on the basis of the appearance of hemorrhage limited to the prepontine and/or perimesencephalic cisterns on computed tomography (CT) scans in the absence of an aneurysm on cerebral angiography [1].

Despite the fact that aneurysmal SAH and BPH are the respective leading diagnoses in spontaneous SAH with and without an identifiable point of origin [1], to the best of the authors' knowledge, no cases of both vascular pathologies occurring in the same individual have been previously reported. Herein we present the case of a patient with aneurysmal SAH followed five years later by BPH.

## Case Presentation

**Figure 1:**
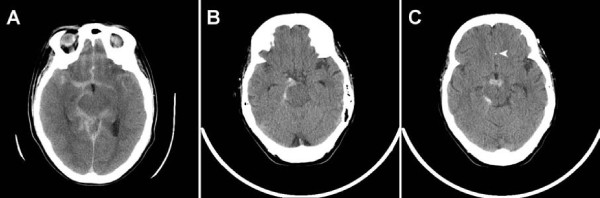
**The patient's non-contrasted head computed tomography scan (A) at the time of her initial aneurysmal subarachnoid hemorrhage admission and (B and C) subsequent non-aneurysmal subarachnoid hemorrhage revealing perimesencephalic hemorrhage**. The superior aspect of the coil mass from her previously treated anterior communicating artery aneurysm, remote from the new non-aneurysmal subarachnoid hemorrhage, is noted (C, arrowhead).

**Figure 2:**
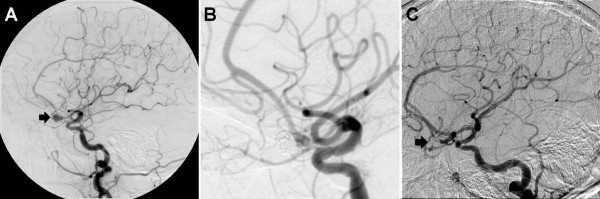
**(A) The patient's initial diagnostic angiogram at the time of aneurysmal subarachnoid hemorrhage**. A 6 mm anterior communicating artery aneurysm is shown (arrow). The aneurysm was treated in the same setting with endovascular coiling. A follow-up diagnostic angiogram obtained eight months after aneurysmal subarachnoid hemorrhage shows complete obliteration (B, arrow). At the time of her perimesencephalic hemorrhage five years later, a diagnostic angiogram reveals the previously coiled anterior communicating artery aneurysm (C, arrowhead); no residual filling of the aneurysm was noted, and no other vascular abnormalities were seen.

The patient was a Caucasian, non-smoking 51-year-old woman with insulin-dependent diabetes and hypertension who initially presented at the age of 46 with acute-onset ictal headache, meningismus and emesis (Hunt/Hess grade I). A non-contrasted head CT scan revealed SAH in an aneurysmal pattern (Figure 1A). She underwent cerebral angiography, which revealed a 6 mm anterior communicating artery (Acomm) aneurysm (Figure 2A) that was treated with endovascular coiling in the same setting. At the end of the procedure, a 0.5 mm residual was noted at the base of the aneurysm that incorporated the anterior cerebral arteries and was not treated (not shown). Follow-up angiography eight months later showed complete obliteration (Figure 2B). The patient's hospital course was complicated by vasospasm, which was treated with hypervolemia, hypertension and intra-arterial nicardipine, as well as cerebral salt wasting, which was treated with sodium and volume supplementation. She was ultimately discharged to home and returned to work six weeks later with no residual neurologic deficits. After her eight-month posthemorrhage angiogram, she was lost to neurosurgical follow-up. Of note, the patient developed peritoneal dialysis-dependent renal failure three years later. A renal ultrasound did not demonstrate evidence of polycystic kidney disease (not shown).

Prior to the patient's current admission (five years after her aneurysmal SAH), the patient had experienced three days of intermittent nausea and vomiting. On the day of admission, she developed an acute-onset severe headache with worsened nausea and emesis. On admission, the patient's condition was Hunt/Hess grade II with an initial non-contrasted head CT scan demonstrating perimesencephalic SAH in the prepontine, interpeduncular, ambient and crural cisterns (Figure 1B and 1C). Of note, no hemorrhage was noted adjacent to the previously treated Acomm aneurysm (Figure 1C, arrowhead). Although there was no evidence of thrombocytopenia or other coagulopathy on her admission laboratory testing, she was taking clopidogrel and received a pool of platelets. A toxicology screen revealed no evidence of sympathomimetic use. Diagnostic cerebral angiography did not reveal any new aneurysms or vasculopathy and showed the previously treated aneurysm to be stable with no residual (Figure 2C). The diagnostic angiogram also demonstrated patent cerebral venous sinuses without evidence of thrombosis or stenosis. Given the lack of vasculitic changes on the angiogram, further workup for vasculitis was not performed. The patient had a follow-up angiogram eight days later that again failed to show any source for the hemorrhage, consistent with BPH (not shown). The patient had an uneventful hospital course and was discharged to home in good condition on post-bleed day 10. Follow-up magnetic resonance imaging and angiography performed six months later demonstrated no vascular abnormalities (not shown).

## Discussion

Despite the recent identification of BPH as a distinct vascular pathology [2], it is now purported to be a primary etiology of non-aneurysmal SAH [1,3]. In contrast to aneurysmal SAH, BPH, for which some authors have proposed the term *pre-truncal non-aneurysmal hemorrhage *[4], is thought to arise from multiple possible non-arterial sources [2,5]. Previous studies have reported that patients with BPH have normal life expectancies and are not at risk for re-bleeding [1]. Other studies have noted some, albeit reduced, post-hemorrhage complications compared to aneurysmal SAH, including vasospasm, post-hemorrhagic hydrocephalus and death [6].

The patient in this case had BPH that was both temporally and spatially remote from her previous aneurysmal SAH. Other than her general risk factors for SAH, which include female sex, hypertension and previous ruptured aneurysm, the literature offers little insight regarding a probable underlying pathology that could account for both of these hemorrhages. There have been previous reports of BPH occurring in individuals with various vascular pathologies, including ischemic stroke [5] and venous stenosis or thrombosis [7,8], but none that the authors know of in the setting of a previously ruptured intra-cranial aneurysm. Rebleeding from BPH has been reported only once, although it occurred after early anti-coagulation [9].

The location of the patient's aneurysmal rupture was fortuitous as it related to her subsequent BPH. It was evident that the location of her perimesencephalic hemorrhage did not extend to the region of her previously coiled Acomm aneurysm and most likely arose from a separate process. This permitted the diagnosis of BPH to be made and a less aggressive treatment course to be pursued. Her treatment would have been significantly more complicated had her aneurysm been in the posterior circulation within the region of her BPH. In this setting, a diagnosis of BPH would have been difficult to justify, and she possibly would have undergone attempts at either recoiling or even open clipping of a suspected unsecured aneurysm.

Multiple previous case series have attested to the relatively benign course of perimesencephalic hemorrhage [2,3,6,10]. It would not be unreasonable, however, to posit that the currently presented patient may have fared worse than expected, given her previous aneurysmal SAH. Fortunately, this was not the case. There is no evidence that the first bleeding event rendered her more susceptible to a second, less severe event. It is unknown what effect BPH occurring shortly after aneurysmal SAH or in someone with a poorer grade injury would have on neurologic outcome.

## Conclusions

This work represents the first report of both aneurysmal SAH and non-aneurysmal BPH occurring in the same individual. The diagnosis of BPH may be complicated by previous aneurysmal SAH. The expected good prognosis associated with BPH does not appear to be altered by a previous episode of aneurysmal SAH. To those in the fields of neurology and neurosurgery, this case serves as an important reminder that in patients with a history of previous aneurysmal SAH, subsequent episodes of SAH need to be fully investigated because they may be attributable to an entirely different pathology.

## Consent

Written informed consent was obtained from the patient for publication of this case report and accompanying images. A copy of the written consent is available for review by the Editor-in-Chief of this journal.

## Competing interests

The authors declare that they have no competing interests.

## Authors' contributions

RS was responsible for the initial care of the patient, along with the conception and writing of the manuscript. DK and HK were responsible for patient management and workup for benign perimesencephalic hemorrhage. MH was responsible for the patient's original aneurysmal management and supervised her case on her subsequent admission. All authors read and approved the final manuscript.
